# Mesenchymal Stem Cell-Derived Molecules Reverse Fulminant Hepatic Failure

**DOI:** 10.1371/journal.pone.0000941

**Published:** 2007-09-26

**Authors:** Biju Parekkadan, Daan van Poll, Kazuhiro Suganuma, Edward A. Carter, François Berthiaume, Arno W. Tilles, Martin L. Yarmush

**Affiliations:** 1 Center for Engineering in Medicine and Surgical Services, Massachusetts General Hospital, Harvard Medical School and the Shriners Hospitals for Children, Boston, Massachusetts, United States of America; 2 Harvard-Massachusetts Institute of Technology Division of Health Sciences and Technology, Cambridge, Massachusetts, United States of America; 3 Department of Surgery, University Medical Center, University of Utrecht, Utrecht, The Netherlands; Beijing Institute of Infectious Diseases, China

## Abstract

Modulation of the immune system may be a viable alternative in the treatment of fulminant hepatic failure (FHF) and can potentially eliminate the need for donor hepatocytes for cellular therapies. Multipotent bone marrow-derived mesenchymal stem cells (MSCs) have been shown to inhibit the function of various immune cells by undefined paracrine mediators *in vitro*. Yet, the therapeutic potential of MSC-derived molecules has not been tested in immunological conditions *in vivo.* Herein, we report that the administration of MSC-derived molecules in two clinically relevant forms-intravenous bolus of conditioned medium (MSC-CM) or extracorporeal perfusion with a bioreactor containing MSCs (MSC-EB)-can provide a significant survival benefit in rats undergoing FHF. We observed a cell mass-dependent reduction in mortality that was abolished at high cell numbers indicating a therapeutic window. Histopathological analysis of liver tissue after MSC-CM treatment showed dramatic reduction of panlobular leukocytic infiltrates, hepatocellular death and bile duct duplication. Furthermore, we demonstrate using computed tomography of adoptively transferred leukocytes that MSC-CM functionally diverts immune cells from the injured organ indicating that altered leukocyte migration by MSC-CM therapy may account for the absence of immune cells in liver tissue. Preliminary analysis of the MSC secretome using a protein array screen revealed a large fraction of chemotactic cytokines, or chemokines. When MSC-CM was fractionated based on heparin binding affinity, a known ligand for all chemokines, only the heparin-bound eluent reversed FHF indicating that the active components of MSC-CM reside in this fraction. These data provide the first experimental evidence of the medicinal use of MSC-derived molecules in the treatment of an inflammatory condition and support the role of chemokines and altered leukocyte migration as a novel therapeutic modality for FHF.

## Introduction

Clinical trials testing the efficacy of bioartificial liver support in treating fulminant hepatic failure (FHF) have provided some promising results, yet the current generation of devices has not demonstrated sufficient efficacy and reliability for routine use, primarily due to a lack of a functionally stable, human hepatocyte source [1]. We have previously shown that immunomodulation via interleukin-1 receptor antagonism in the form of: (a) the recombinant protein, (b) adenoviral vector gene therapy, or (c) transfected hepatocytes seeded in an extracorporeal device, can provide a survival benefit in an animal model of FHF [2], [3]. However, due to concerns associated with gene transfer *in vivo* or *ex vivo* and the expense of recombinant protein production, we sought to identify a natural human “cellular equivalent” of immunomodulation that could be incorporated into bioartificial liver assist devices.

Within the bone marrow space multipotent stromal cells, also referred to as mesenchymal stem cells (MSCs), are known to be the precursor cell for stromal tissues that support hematopoiesis [4]. The immunomodulatory function of MSCs was first reported after it was observed that they could evade immunosurveillance after cell transplantation [5]. This ability of MSCs to alter an immune response has been exploited for therapeutic purposes as demonstrated by the case of a patient suffering from steroid-refractory graft-versus-host disease who was successfully treated by the infusion of haploidentical MSCs [6]. I*n vitro* studies have subsequently shown that MSCs actively inhibit the function of several immune cells through secreted cytokines, growth factors and enzymatic action, although controversy exists on the identity of the responsible mediators [7], [8], [9], [10], [11]. The fortification of the soluble microenvironment by MSCs can also affect non-hematopoietic cells as well. MSCs used for cellular cardiomyoplasty after an ischemic event revealed that MSC-derived soluble molecules inhibited hypoxia-induced apoptosis of cardiomyocytes during the acute phase of injury resolution [12], [13]. Taken together, these studies indicate that MSCs can independently affect immune and tissue cells by paracrine means.

On this basis, we hypothesized that the paracrine function of MSCs may be of therapeutic value when used in the setting of acute organ failure, wherein parenchymal cell loss is integrated with a local and systemic inflammatory response. Here, we report a significant survival benefit in the treatment of FHF after intravenous delivery of MSC biomolecules in the form of bolus injections of conditioned medium (MSC-CM) or a MSC-based extracorporeal bioreactor (MSC-EB). The survival benefit of MSC-CM was a function of the cell mass used for media conditioning and was found to have an optimum. Histological analysis of liver tissue after MSC-CM treatment showed a remarkable reduction in infiltrating leukocytes as well protection of hepatobiliary pathological changes. In addition, we show that MSC-CM actively promotes the emigration of adoptively transferred leukocytes from the injured liver. Finally, we performed a proteomic screen of the MSC secretome and detected high levels of potent chemotactic cytokines. Fractionation of MSC-CM based on affinity to heparin sulfate, a known ligand for all chemokines, revealed that the therapeutic activity of MSC-CM was restricted to the heparin bound fraction, further supporting the role of chemokines as responsible mediators.

## Results

### MSC-derived components reverse FHF

**Figure 1:**
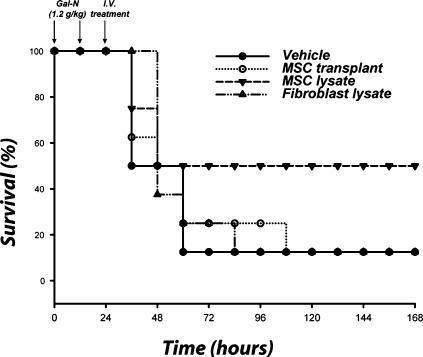
Infusion of MSC lysate provides a survival trend in an animal model of FHF. Sprague-Dawley rats were administered lethal intraperitoneal injections of a hepatotoxin. Animals were treated with i.v. injections of MSCs, or MSC lysates from the same cell mass (2×10^6^ cells). Controls received vehicle or NIH 3T3-J2 fibroblast components. Kaplan-Meier survival analysis of Gal-N administered rats treated with cell transplants or lysates. Time points of interventions are stated above survival plots. Results are cumulative data of two independent experiments (N = 8 per each group) using different batches of MSCs. *P*-value determined by Log Rank Test.

We first assessed various MSC treatment modalities such as cell transplantation, delivery of cellular lysates or conditioned medium to assess the most efficacious therapy. Sprague-Dawley rats were intraperitoneally administered a total of two injections of 1.2 g/kg of a hepatotoxin, D-galactosamine (Gal-N), each separated by 12 hours [14]. With this regimen of liver failure induction, we have previously shown that death occurs 36–72 hours after injection of the first dose and is associated with significant hepatocellular necrosis concomitant with leukocyte infiltration and an increase of inflammatory cytokines in liver tissue. Animals were treated 24 hours after FHF induction with intravenous injections of whole cells or cell lysates. No significant benefit was seen after the intravenous infusion of 2×10^6^ human MSCs 24 hours post-induction, which is most likely due to poor engraftment, entrapment in the alveolar capillary bed and/or immune rejection of the cells (Fig. 1). In contrast, treatment with cellular lysates, derived from the same cell mass used for transplantation, showed an increased survival trend compared to vehicle (*P*<0.47) and fibroblast lysate (*P*<0.36) controls.

**Figure 2:**
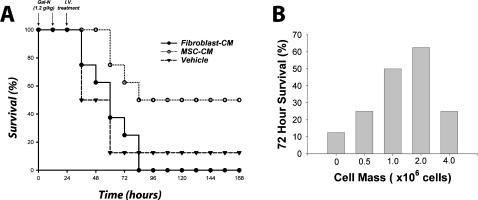
MSC-CM reverses FHF in a cell mass-dependent manner. (A) Kaplan-Meier survival analysis of Gal-N administered rats treated with concentrated MSC-CM. (B) Dose response of animal survival 72 hours after liver failure induction as a function of MSC mass from which MSC-CM was derived. Controls received vehicle or fibroblast conditioned medium (fibroblast-CM). Time points of interventions are stated above survival plots. Results for both panels are cumulative data of two independent experiments using different batches of MSC-CM (N = 8 per each group). *P*-value determined by Log Rank Test.

We then determined if the efficacy observed with lysates could be reproduced by using the secreted molecules from MSCs. A longitudinal study using MSC-CM from the same cell mass (i.e., 2×10^6^ human MSCs) revealed a distinct survival benefit compared to vehicle (*P*<0.032) and fibroblast (*P*<0.026) concentrated medium (Fig. 2A). In addition, we monitored 72 hour survival of FHF-induced rats as a function of MSC mass from which medium was conditioned (Fig. 2B). Interestingly, the effect of MSC concentrate was abrogated at higher cell masses indicating a therapeutic window of effectiveness. Moreover, the observation that xenogeneic MSC lysates and supernatants decreased animal mortality suggests that these factors can cross species barriers.

### Combined metabolic and secretory function in MSC-EB support provide hepatoprotection and survival benefit

**Figure 3:**
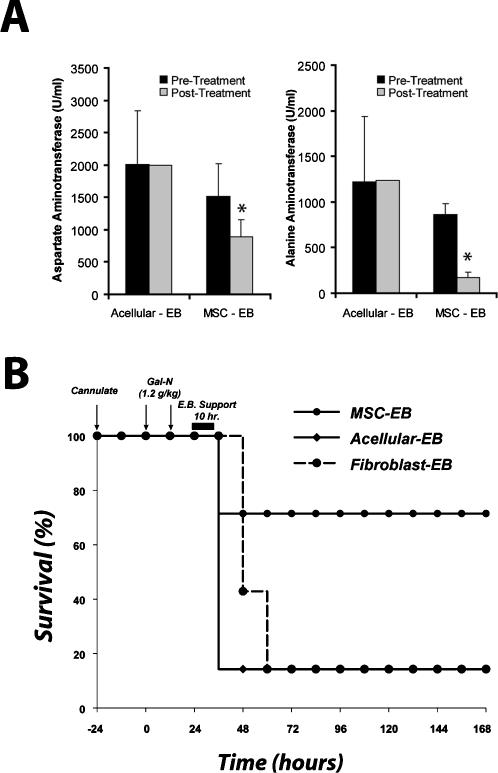
MSC-EB support reduces liver injury biomarkers and increases survival. Animals were treated with an MSC-EB, using a 3T3 fibroblast-based bioreactor (fibroblast-EB) and an acellular bioreactor (acellular-EB) as controls. (A) Serum biomarkers of liver injury, aspartate aminotransferase and alanine aminotransferase preceding and 24 hours after treatment with a MSC-EB (n = 5) or an acellular-EB (n = 3). Due to mortality, n = 1 in the acellular group after treatment. (B) Kaplan-Meier survival analysis of Gal-N administered rats treated with EBs. Time points of interventions are stated above survival plots. Each result for (B) was an independent experiment using different batches of MSCs. *P*-value determined by student's t-test analysis for panel (A). *P*-value determined by Log Rank Test for panel (B).

Based on these results, we then developed a MSC-EB to combine the effectiveness of intracellular and secreted molecules of MSCs in a single device. Animals were treated 24 hours after FHF induction with a human MSC-EB connected to the systemic circulation of the animal. Bioreactors seeded with fibroblasts (fibroblast-EB) and acellular (acellular-EB) bioreactors served as controls. After 10 hours of extracorporeal perfusion, animals were taken off assist support and monitored for survival. Plasma was obtained at the start of, and 24 hours after, bioreactor treatment and analyzed for hepatocyte enzyme release. Liver serologies, including aspartate aminotransferase (*P*<0.02) and alanine aminotransferase (*P*<0.001) were improved in animals treated with the MSC-EB (Fig. 3A). These data demonstrate a hepatoprotective effect of device therapy as shown by the reduction in biochemical markers of hepatocyte death. More importantly, 71% of animals treated with the MSC-EB survived, compared to 14% (Fig. 3B) in both acellular (*P*<0.037) and fibroblast controls (*P*<0.05).

### MSC-CM therapy inhibits panlobular leukocyte invasion, bile duct duplication and hepatocellular death

**Figure 4:**
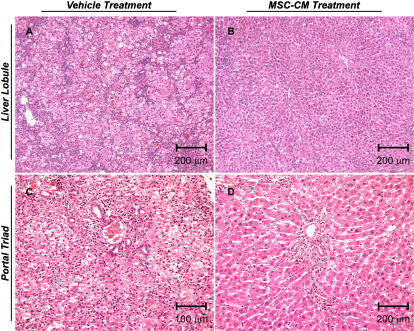
MSC-CM treatment inhibits immune cell infiltration and hepatobiliary cell change in Gal-N injured liver tissue. Representative H&E histology sections of liver tissue from Gal-N injured rats 36 hours post-treatment with vehicle (A,C) or MSC-CM (B, D). Scale bars are indicated on the micrographs. Images (A, B) and (C,D) are captured 10× and 20× magnification, respectively.

In order to determine histopathological changes after MSC-CM therapy we used a sub-lethal regimen Gal-N to induce acute liver injury, thereby ensuring survival in our control-treated group for comparison. It should be noted that even at this Gal-N dose, mortality occurred in a vehicle-treated group (N = 1) confirming that the extent of injury in this model can still be fatal. Gal-N injured rats were treated with vehicle (N = 4) or MSC-CM (N = 4) 24 hours after injury and their livers were harvested 36 hours thereafter for pathological analysis. Microscopic evaluation of liver tissue from vehicle treated rats revealed profound hepatocellular apoptosis, bile duct duplication and panlobular mononuclear leukocyte infiltration with cytoplasmic vacuolization and severe distortion of tissue architecture (Fig. 4A,C). MSC-CM treated rats showed no signs of disseminated inflammation (Fig. 4B), although minor periportal infiltration with edema and fibrin deposition consistent with tissue repair was observed (Fig. 4D).

### MSC-CM treatment alters immune cell migration to the liver

**Figure 5:**
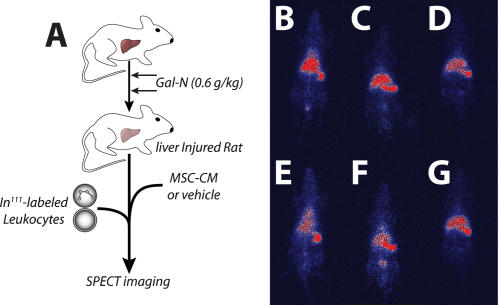
Alteration in leukocyte migration after MSC-CM treatment. (A) Experimental design of adoptive transfer study. Gal-N injured rats were treated with vehicle or MSC-CM followed by infusion of In^111^-labeled leukocytes. SPECT images were acquired at t = 0, 3, and 24 hr. for MSC-CM (B–D) and vehicle (E–G) treated rats, respectively.

We hypothesized that one potential explanation for the lack of panlobular leukocyte infiltration may be that MSC-CM treatment may divert immune cell migration away from an inflamed, target organ. To test this theory, we adoptively transferred radiolabeled leukocytes, directly after MSC-CM or vehicle treatment, into Gal-N (0.6 g/kg) injured rats and monitored leukocyte trafficking using single photon emission computed tomography (SPECT) over time (Fig. 5A). Qualitatively, more leukocytes were seen migrating to the liver in vehicle treated animals over time (Fig. 5E–G). In contrast, there was a distinct decrease in signal intensity in the liver of MSC-CM treated animals over time (Fig. 5B–D). These results suggest that there was a selective pressure upon leukocytes to emigrate from the liver due to MSC-CM, unlike control conditions where leukocytes eventually migrated to the injured organ. These data support our hypothesis that altered leukocyte migration may be a potential target of MSC-CM therapy, however other explanations cannot be ruled out without more comprehensive investigations.

### MSC-CM is composed of many chemokines that correlate with therapeutic activity

**Figure 6:**
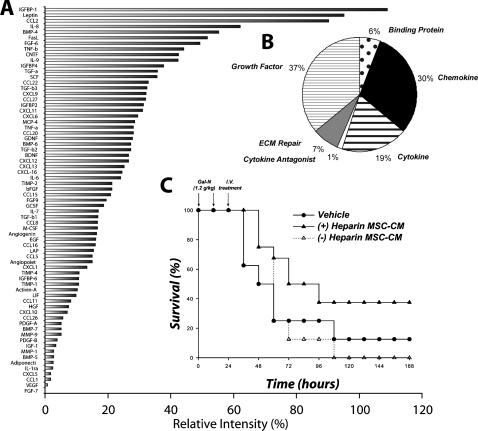
MSC-CM is composed of high levels of chemokines that correlate with survival benefit seen in FHF. Serum-free MSC-CM was analyzed using an antibody array for 174 specified proteins. (A) Densiometry of spotted antibody array results. Data are presented as spot intensity relative to the negative control and normalized to positive control. (B) Pie chart showing cluster analysis of MSC secreted proteins based on reported function. MSC-CM was fractionated over a heparin-agarose column into heparin bound and unbound fractions. (C) Kaplan-Meier survival analysis of Gal-N administered rats treated with the (+) heparin MSC-CM and (−) heparin MSC-CM. Time points of interventions are stated above survival plots. Results for (C) are cumulative data of two independent experiments using different batches of MSC-CM (N = 8 per each group). *P*-value determined by Log Rank Test.

In an effort to understand the molecular mediators of the observed effects of MSC therapy, we examined MSC-CM using a high-density protein array. MSC-CM contained 69 of the 174 proteins assayed (Fig. 6A), which included a broad spectrum of molecules involved in immunomodulation and liver regeneration. Cluster analysis revealed that a large fraction (30%) of MSC-CM was composed of chemokines (Fig. 6B), many of which were expressed at very high relative levels. We decided to fractionate MSC-CM based on functionality using affinity-based methods rather than other arbitrary molecular criteria such as size or hydrophobicity. MSC-CM was passed over an affinity column impregnated with heparin sulfate, a known ligand for all chemokines and separated into bound and unbound fractions. Each fraction was infused into FHF-induced rats with overall survival as the study endpoint. We observed that the therapeutic activity of MSC-CM was restricted to the heparin bound fraction, providing a strong correlation between chemokines and the survival benefit after MSC-CM infusion in FHF-induced rats (Fig. 6C).

## Discussion

Our findings demonstrate that MSC-derived molecules can protect against hepatocyte death and increase survival in Gal-N induced FHF. Specifically, we have shown that an intravenous bolus of MSC-CM during active disease can reverse organ failure. The efficacy of MSC-CM was found to be a function of the cell mass from which media were conditioned suggesting important pharmacological aspects of this treatment. Treatment with a MSC-EB, which combined both the secretory and metabolic functions of MSCs in a single device, provided the greatest benefit in survival and potentially illustrates a platform to study stem cell function and the bone marrow microenvironment *ex vivo*. These results are significant because we have identified a non-hepatic source of human cells with minimal metabolic demands that can be expanded to clinical scale for liver assist devices and shown that MSC treatment can cross species barriers.

Histological evaluation of liver tissue after MSC-CM treatment provided initial insight into the cellular targets of therapy. A striking reduction in mononuclear leukocytes was seen post-treatment, suggesting that MSC-CM may inhibit the ability of immune cells to invade and/or function within the injured tissue. Using SPECT imaging of adoptively transferred leukocytes, we provide evidence showing altered immune cell migration after MSC-CM treatment supporting our hypothesis that MSC-CM modulated the immune response to organ injury. Interestingly, it appears that MSC-CM did not divert leukocyte trafficking from the injured site initially, but rather induced a selective emigration of homed leukocytes out of the organ. In addition, we observed prevention of hepatocyte apoptosis and inhibition of bile duct duplication after MSC-CM treatment. Further investigation is needed to determine if these are direct or indirect effects of MSC-CM as well as the temporal sequence of histological changes to evaluate which processes are the primary targets of MSC-CM and which are merely secondary effects.

Proteomic analysis of MSC-CM revealed a broad spectrum of molecules involved in immunomodulation and liver regeneration. Powered by our knowledge that MSC-CM specifically alters leukocyte migration, we first looked at chemotactic cytokines as potential mediators of the therapeutic effect of MSC-CM. Approximately 30% of MSC-CM was composed of relatively high levels of potent chemokines that, when fractionated based on heparin binding affinity, correlated with the therapeutic activity of MSC-CM. We are currently testing individual chemokines and combinations thereof to demonstrate a casual relationship. Moreover, since any positively charged molecule can ionically interact with heparin sulfate, we cannot rule out other heparin binding compounds such hepatocyte growth factor within the screen of MSC-CM.

In conclusion, we describe the first use of the secreted and metabolic functions of MSCs to derive a new class of immunotherapeutics. Whether this strategy of MSC-CM or MSC-EB therapy is a relevant means of treatment for other organ failure and inflammatory conditions should be experimentally explored.

## Materials and Methods

Materials were purchased from Sigma-Aldrich, St. Louis, MO unless otherwise stated.

### Cell Culture

Human MSCs were isolated from commercially available bone marrow aspirates (Clonetics-Poietics, Walkersville, MD) and grown and characterized as previously reported [15]. Cells were used for experiments during passages 3-7. NIH 3T3-J2 fibroblasts were a kind gift from Dr. Howard Green and cultured according to donor's protocols.

### Preparation and delivery of cells, cell lysates and conditioned medium

Cellular lysates were prepared by sonication (VWR Scientific, West Chester, PA). The dose of sonicated cells administered was 2×10^6^ cells per subject, which represented the same cell mass used for extracorporeal bioreactor or cell transplant experiments. Conditioned medium was prepared by collecting serum-free medium (supplemented with 0.05% bovine serum albumin to prevent protein aggregation) after 24 hour culture of different cell masses. The majority of experiments were performed with the optimal cell mass of 2×10^6^ cells. The medium was then concentrated, approximately 25 fold, using ultrafiltration units (Amicon Ultra-PL 3, Millipore, Bedford, MA, USA) with a 3 kDa molecular weight cut-off. For fractionation experiments, concentrated medium was passed over a heparin-agarose column and the flow-through and eluted fractions were collected and reconcentrated using the same ultrafiltration system. A total volume of 500 µl containing cells, cellular lysates, vehicle (PBS) only or 900 µl of conditioned medium was infused in the penile vein 24 hours after induction of FHF using the same anaesthesia protocol described for the cannulation procedure.

### Liver Failure Induction and Extracorporeal Bioreactor Support

The induction of fulminant hepatic failure and extracorporeal device operation is previously reported [2]. Briefly, male Sprague-Dawley rats weighing between 280 and 370 grams were anaesthetized using intraperitoneal injections of ketamine and xylazine at 110 and 0.4 mg/kg, respectively. The left carotid artery and right jugular vein were cannulated and the animal was placed in a metabolic cage. Twenty-four hours later, 1.2 g/kg Gal-N freshly dissolved in physiological saline and adjusted to pH 7.3 with 1 N NaOH was injected i.p., followed by a second equal injection 12 hours later. Twenty-four hours after the first injection of Gal-N, the arterial and venous lines were connected to an extracorporeal circuit. Plasma was separated using a plasma separator (MicroKros, pore size 0.2 micron). Plasma was perfused through the polycarbonate, flat-plate bioreactor and subsequently reunited with the cellular components of the blood and returned to the animal. The extracorporeal bioreactor was operated for 10 hours. Animals that died during reactor operation and failed to receive adequate treatment (MSC-EB, N = 3 and Fibroblast-EB, N = 2) were censored from analysis. Animal survival was monitored every 12 hours. Plasma or whole blood was analyzed for liver injury biomarkers using a microfluidic metabolic assay (Picollo, Abaxis, Union City, CA).

### Liver Histology

Liver tissue was harvested from rats induced with a sub-lethal regimen of Gal-N (0.6 g/kg), 36 hours after treatment with MSC-CM. Tissue was fixed in 10% buffered formalin, embedded in paraffin, sectioned to 6-µm thickness, and stained with hematoylin and eosin.

### Adoptive transfer of radiolabeled leukocytes

Leukocytes were isolated from whole rat blood by NH_4_Cl erythrocyte lysis. Cells were pelleted, washed once with PBS and resuspended in 0.9% saline containing the In^111^ oxine isotope (GE Healthcare Biosciences Corp., Piscataway, NJ). Cells were labeled at 92% efficiency with high viability. Approximately 15×10^6^ cells were infused into the penile vein of Gal-N injured (0.6 g/kg) directly after treatment with vehicle or MSC-CM. SPECT images were captured using a M.CAM gamma camera setup (Siemens Medical Systems, Malvern, PA) at 0, 3 and 24 hours after leukocyte infusion.

### Protein array of MSC supernatants

Supernatants were prepared by collecting serum-free medium after 24 hour culture of approximately 2×10^6^ MSCs. These were analyzed for a panel of specified proteins using an antibody array (RayBio Human Cytokine Antibody Array C Series 2000, RayBiotech Inc., Norcross, GA) as specified by the vendor.
